# Parents’ life satisfaction prior to and following preterm birth

**DOI:** 10.1038/s41598-023-48582-8

**Published:** 2023-12-01

**Authors:** Robert Eves, Nicole Baumann, Ayten Bilgin, Daniel Schnitzlein, David Richter, Dieter Wolke, Sakari Lemola

**Affiliations:** 1 Fakultät Für Psychologie Und Sportwissenschaft, Abteilung Für Psychologie, Universität Bielefeld, 10 01 31, Bielefeld, Germany; 2 Department of Psychology, Lifespan Health and Wellbeing Group, University of Warwick, Coventry, UK; 3 Department of Health Sciences, University of Leicester, Leicester, UK; 4 Turner Institute for Brain and Mental Health, School of Psychology Sciences, Monash University, Melbourne, Australia; 5 Department of Psychology, University of Essex, Colchester, Essex UK; 6 Leibniz University of Hannover, Hannover, Germany; 7 IZA Bonn, Bonn, Germany; 8 German Institute for Economic Research, (DIW Berlin, Deutsches Institut Für Wirtschaftsforschung E.V.), Berlin, Germany

**Keywords:** Human behaviour, Paediatric research

## Abstract

The current study tested whether the reported lower wellbeing of parents after preterm birth, relative to term birth, is a continuation of a pre-existing difference before pregnancy. Parents from Germany (the German Socio-Economic Panel Study, N = 10,649) and the United Kingdom (British Household Panel Study and Understanding Society, N = 11,012) reported their new-born’s birthweight and gestational age, subsequently categorised as very preterm or very low birthweight (VP/VLBW, < 32 weeks or < 1500 g), moderately/late preterm or low birthweight (MLP/LBW, ≥ 32 weeks and < 37 weeks/≥ 1500 g and < 2500 g), or term-born (≥ 37 weeks and ≥ 2500 g). Mixed models were used to analyse life satisfaction, an aspect of wellbeing, at four assessments-two years and six months before birth and six months and two years afterwards. Two years before birth, satisfaction of prospective term-born, MLP/LBW, or VP/VLBW mothers did not significantly differ. However, mothers of VP/VLBWs had lower satisfaction relative to mothers of term-borns at both assessments post-birth. Among fathers, satisfaction levels were similarly equivalent two years before birth. Subsequently, fathers of VP/VLBWs temporarily d﻿iffered in satisfaction six months post-birth relative to fathers of term-borns. Results indicate that parents’ lower life satisfaction after VP/VLBW birth is not a continuation of pre-existing life satisfaction differences.

## Introduction

Both moderately to late preterm birth or low birthweight (≥ 32 and﻿ <37 weeks gestation or < 2500 g; MLP/LBW), and very preterm birth or very low birthweight especially (< 32 weeks or < 1500 g; VP/VLBW), are associated with increased risk of mortality and morbidity^1^. Relatedly, VP/VLBWs’ average stay in neonatal intensive care units (NICU) is two months^2,3^, which is associated with reduced wellbeing among their parents^4–9^. Subsequently, at up to 12 years after birth, parents of preterms show decreased wellbeing and increased stress levels^8,10,11^, with mothers more affected than fathers^4,7^. Furthermore, in a follow-up study of mothers of VP/VLBWs especially, increased distress was reported at two, seven and 13 years after birth^6^. Parents may therefore struggle to overcome the initial adversity associated with preterm birth as they are at a higher risk for having post-traumatic stress symptoms^12^, however research findings here are somewhat inconsistent^13^. In addition, taking care of a VP/VLBW child, who has a higher risk of developmental delay^14^, may increase the reported long term burden felt by parents^15^. Thus, for parents of MLP/LBWs or VP/VLBWs, and in particular for mothers of VP/VLBWs, research indicates reduced levels of wellbeing during their offspring’s childhood.

An important aspect of subjective wellbeing is general life satisfaction^16^, which has been consistently shown to have a high correlation with fewer mental health problems^17–19^, and better physical health^16^. As a general trend, a transient increase in general life satisfaction has been reported for parents in Britain and Germany in the years directly before and after childbirth, before returning back to pre-pregnancy levels^20,21^. Although potentially true for the majority of the population, research also suggest that this transition to parenthood may differ depending on the parent’s sex, age, education level, or whether the parent has a migrant background^21,22^. What is currently unknown, is whether the course of life satisfaction differs for parents of children born VP/VLBW or MLP/LBW. Due to the aforementioned studies showing mental health differences post-birth, parents of VP/VLBWs or MLP/LBWs may differ from parents of term-borns by showing a transition to parenthood with either a smaller rise, no rise, or even a deterioration in life satisfaction surrounding birth. Alternatively, it has been hypothesised that increased stress and lower life satisfaction may be a precursor of preterm birth^23^, that is prenatal stress and financial hardship increases the risk of preterm birth occurring^23–25^. It is therefore possible that life satisfaction is already low before pregnancy for future parents of preterm children. Thus, any subsequent association found between preterm birth and parents’ lower life satisfaction would not be due to preterm birth per-se but rather a continuation of pre-existing lower life satisfaction. Similarly, many factors that have been associated with lower life satisfaction, such as higher age, lower income, lower education, and migrant background^17,26,27^, have also been associated with increased risk of preterm birth^28,29^. Overall, the idea of a stable difference between parents of preterms or term-borns, before and after the birth, would be in line with results of general population studies showing postpartum depression is highly associated with pre-partum depression^30^.

This study aims to answer two main research questions. First, do future parents of MLP/LBW or VP/VLBWs already have lower life satisfaction than parents of term-borns, up to two years before birth? Second, is the course of life satisfaction from two years before till two years after birth different for parents of MLP/LBWs or VP/VLBWs when compared to parents of term-borns? To answer these questions, data from two large population representative panel studies from Germany and the United Kingdom are studied. These studies both include repeated assessments of parental life satisfaction before and after birth, which allows to check the robustness of our findings. This resulted in the following hypotheses:

1. There will be a difference in life satisfaction two years before birth between future parents of VP/VLBW, MLP/LBW and term born infants, tested separately in mothers and fathers.
2. The changes in life satisfaction of parents before and after birth will differ depending on whether the infant is born VP/VLBW, MLP/LBW, or term born, tested separately in mothers and fathers.

## Methods

### Participants and procedure

This study used data from two international panel studies (1) The German Socio-Economic Panel (SOEP; Version 36)^31^ and (2) the combined datasets of the British Household Panel Study and the Understanding Society study (BHPS/USS)^32^. The SOEP is a random sample of representative households in Germany. While initiated in 1984, it has been annually asking parents about gestation and birthweight of new-borns since 2003, meaning the included infants ranged in birthyear from 2002 to 2019.

Analyses were also performed on data from the United Kingdom. The BHPS is a nationally representative study which started in 1991, collecting information about gestation and birthweight for newborns from 1999 until the final wave in 2008. In 2008, the BHPS was absorbed into the USS study, which has released ten data waves. For this analysis, all results are simply referred to as BHPS/USS data and are the combination of waves 9–18 of the BHPS and waves 1–10 of the USS, meaning the included infants ranged in birthyear from 1997 to 2019^32^. Further information about the SOEP can be found in Goebel et al.^31^ and in Buck and McFall for the BHPS/USS^33^.

In each study, all births with valid data on either birthweight or gestational age were identified. We then categorised births into three groups—VP/VLBW (VP < 32 weeks and/or VLBW < 1500 g), MLP/LBW (MLP < 37 weeks and/or LBW < 2500 g) but not VP/VLBW, or term born (birthweight ≥ 2500 and gestation ≥ 37 weeks). When a parent had multiple children, just one birth was considered for how it influenced life satisfaction. The decision of which birth was made by ranking the births by whether they (1) had valid data for both birthweight and gestation, (2) were VP or VLBW, (3) were MLP or LBW, and finally (4) by birth year. This meant that just one birth would be chosen per parent, with preference given to those with full data, born with lower gestational age or lower birthweight, or if they were the first child reported to the parent.

The outcome variable of self-reported life satisfaction was measured annually in each study, with the exception of the 11th wave of BHPS. For each parent, life satisfaction assessments were only eligible for inclusion if they fell within a certain timeframes relative to the birth and were binned into four specific assessment points. Assessment points were ideally two years before birth (life satisfaction before the pregnancy of the selected birth), six months before birth (life satisfaction during the pregnancy of the selected birth), six months after birth (life satisfaction at the end of the post-natal phase), and two years after birth (long term life satisfaction post birth). Due to births occurring at any time during annual assessments, the first and fourth assessment were eligible for inclusion when they were at most 36 months and at minimum 12 months away from the birth. Similar restrictions were applied on the second and third assessments, being no more than 12 months away from the birth. As the first assessment was before the pregnancy of the child, it was determined that this would reflect “baseline life satisfaction”. Life satisfaction was measured using a single item, which has been found to perform very similarly to multiple item measures of life satisfaction^34^. In the BHPS/USS data, the question was “how dissatisfied or satisfied are you with your life overall?” and answers ranged from one (completely dissatisfied) to seven (completely satisfied). In SOEP, the question was “How satisfied are you with your life, all things considered? (Wie zufrieden sind Sie gegenwärtig, alles in allem, mit Ihrem Leben?)” and it ranged from zero (completely dissatisfied) to ten (completely satisfied). To harmonize life satisfaction, scores were Z standardized separately in each study and separately for mothers and fathers, so that over the four assessments the mean was zero and had a standard deviation of one (M = 0, SD = 1).

A number of covariates were considered. Time invariant binary variables were the child’s sex, whether the child was a singleton or not, whether the child had a known long-standing health condition, whether the child was the first birth reported for the parent, and whether the parent reported a migrant background. Time varying covariates were whether a sibling was born within any of the four assessments, the age of the parent in years, the z standardized net household income for the parent, marital status, and the parent’s highest education level (harmonized into the 1997 International Standard Classification of Education (ISCED) levels of low (levels 1–2), medium (3–4) and high (5 +).

### Ethics approval

For SOEP, ethical permission was granted by the Scientific Advisory Board of DIW Berlin in Germany, and by IRB review at Vanderbilt University (IRB #180737). For BHPS and USS, ethical permission has been repeatedly granted by the University of Essex, with the most recent ethical approval number ETH1920-0123. All studies were performed in accordance with the relevant guidelines and regulations with participants providing informed consent before data collection.

## Data analyses

All analyses were performed in R version 4.1.1^35^, with mixed models performed using the package lme4^36^. Main analyses used all valid data, which was defined as valid life satisfaction data surrounding the sole birth selected per parent and falling within the specifically defined timeframes. Main analyses combined data from the studies but analysed data separately for fathers and mothers while sensitivity analyses in the SOEP specifically removed certain sub-samples (e.g. the refugee specific subsamples), as their transition to parenthood may be particularly stressful^22^. Two between subjects ANOVAs initially analysed parents’ baseline life satisfaction by the child’s birth group variable (VP/VLBW, MLP/LBW, or term-born). A power analysis was conducted on the two ANOVAs with a desired power of 0.8, with an expected moderate standardized mean difference of 0.30 and an expected small difference of 0.15 between the parents of term-borns and the VP/VLBW and MLP/LBW groups respectively. For the baseline satisfaction ANOVAs, life satisfactions scores were Z standardized based solely on the mean and standard deviation of assessment one, two years before birth. For the linear mixed models, as previously noted, scores were Z standardized based on all four assessments as to investigate longitudinal changes. Longitudinal analyses were performed as unbalanced panels, allowing for parents to vary in their number of life satisfaction assessments. Parents may not have all four assessment points due to not being interviewed in the specific timeframes that were subsequently imposed, due to not being recruited before the birth of their child, or due to not taking part in the study at a specific point (e.g. loss of contact/refusal to participate). The multiple assessments per parent were treated as a random effect while the assessment point, birth group, and the interaction between these two factors were treated as fixed effects. In the mixed models, the estimated marginal means of birth groups were calculated at each assessment point. Using the R package emmeans, marginal means were tested for significant differences with a false discovery rate adjustment, akin to a post hoc analysis in an ANOVA^37^. Further sensitivity analyses considered confounding by conducting linear mixed models before and after including the aforementioned covariates or removing specific sub-samples, ran separately in each study and separately for fathers and mothers.

## Results

### Data availability and demographics

In the German Socio-Economic Panel study (SOEP) data, 8611 births were reported in the study period to 6282 mothers. Once combined with eligible life satisfaction data, there were 4754, 865 and 107 mothers of term-born, MLP/LBW, and VP/VLBW infants respectively. Similarly, when the respective fathers were identified, there were 4079, 750 and 94 fathers of term-born, MLP/LBW, or VP/VLBW infants respectively. Further information regarding data availability and participant demographics is shown in Table 1 and Supplementary Table S1.

**Table 1:** German data- SOEP data availability.

	Fathers			Mothers		
	Term Born (N = 4079)	MLP/LBW (N = 750)	VP/VLBW (N = 94)	Term Born (N = 4754)	MLP/LBW (N = 865)	**VP/VLBW** **(N = 107)**
Baseline Life Satisfaction Z Score (2 years before birth)						
Mean (SD)	0.007 (0.98)	−0.023 (1.10)	−0.080 (0.92)	0.001 (0.99)	−0.023 (1.08)	0.142 (0.93)
N within specific timeframe	1628 (40%)	342 (46%)	41 (44%)	1931 (41%)	402 (46%)	46 (43%)
Time from Assessment to Birth (months)						
1st Assessment (Mean (SD))	−23.0 (4.53)	−22.7 (4.93)	−22.9 (4.12)	−23.1 (4.46)	−22.5 (4.81)	−23.9 (4.49)
2nd Assessment (Mean (SD))	−5.64 (3.43)	−5.46 (3.42)	−5.60 (3.22)	−5.62 (3.42)	−5.47 (3.40)	−5.97 (3.08)
3rd Assessment (Mean (SD))	6.19 (3.27)	6.21 (3.24)	6.30 (3.29)	6.21 (3.24)	6.31 (3.26)	5.94 (3.17)
4th Assessment (Mean (SD))	24.0 (4.87)	23.3 (5.11)	23.7 (4.73)	24.1 (4.88)	23.7 (4.97)	24.4 (4.40)
Participants’ number of eligible life satisfaction assessments						
1	1242 (30.4%)	171 (22.8%)	28 (29.8%)	1463 (30.8%)	221 (25.5%)	32 (29.9%)
2	953 (23.4%)	213 (28.4%)	18 (19.1%)	1087 (22.9%)	219 (25.3%)	17 (15.9%)
3	765 (18.8%)	159 (21.2%)	20 (21.3%)	845 (17.8%)	159 (18.4%)	29 (27.1%)
4	1119 (27.4%)	207 (27.6%)	28 (29.8%)	1359 (28.6%)	266 (30.8%)	29 (27.1%)

*SOEP* German socio-economic panel, *MLP/LBW* moderately/late preterm or low birthweight, *VP/VLBW* very preterm/very low birthweight.

In the British Household Panel Study and the Understanding Society study (BHPS/USS) data, 9601 births were reported in the study period to 6670 mothers. Once combined with eligible life satisfaction data, there were 5665, 627 and 105 mothers of term-born, MLP/LBW, and VP/VLBW infants respectively. Similarly, when the respective fathers were identified, there were 4096, 441 and 78 fathers of term-born, MLP/LBW, or VP/VLBW infants respectively. Further information regarding data availability and participant demographics is shown in Table 2 and Supplementary Table S2.

**Table 2:** UK Data- BHPS and USS data availability.

	Fathers			Mothers		
	Term Born (N = 4096)	MLP/LBW (N = 441)	VP/VLBW (N = 78)	Term Born (N = 5665)	MLP/LBW (N = 627)	VP/VLBW (N = 105)
Baseline Life Satisfaction Z Score (2 years before birth)﻿						
Mean (SD)	0.011 (0.99)	−0.0597 (1.01)	−0.178 (1.24)	0.013 (0.99)	−0.083 (1.08)	−0.074 (0.79)
N within specific timeframe	1872 (46%)	226 (51%)	41 (53%)	2674 (47%)	354 (56%)	53 (50%)
Time from Assessment to Birth (months)						
1st Assessment (Mean (SD))	−22.6 (4.92)	−22.7 (5.41)	−22.3 (5.24)	−22.8(5.03)	−22.7(4.94)	−23.2(4.91)
2nd Assessment (Mean (SD))	−5.64 (3.46)	−5.54 (3.45)	−5.43 (3.53)	−5.70 (3.44)	−5.58 (3.46)	−5.87 (3.56)
3rd Assessment (Mean (SD))	6.11 (3.48)	6.08 (3.35)	6.41 (3.46)	6.05 (3.49)	6.14 (3.40)	6.50 (3.43)
4th Assessment (Mean (SD))	23.4 (4.81)	23.4 (4.75)	23.8 (5.09)	23.3 (4.76)	23.1 (4.71)	23.2 (4.09)
Participants’ number of eligible life satisfaction assessments						
1	988 (24.1%)	103 (23.4%)	21 (26.9%)	1222 (21.6%)	124 (19.8%)	24 (22.9%)
2	948 (23.1%)	95 (21.5%)	14 (17.9%)	1252 (22.1%)	123 (19.6%)	15 (14.3%)
3	1071 (26.1%)	122 (27.7%)	19 (24.4%)	1627 (28.7%)	178 (28.4%)	33 (31.4%)
4	1089 (26.6%)	121 (27.4%)	24 (30.8%)	1564 (27.6%)	202 (32.2%)	33 (31.4%)

*BHPS* British Household Panel Stud, *USS* Understanding Society Study, *MLP/LBW* moderately/late preterm or low birthweight, *VP/VLBW* very preterm/very low birthweight.

### Differences in baseline life satisfaction two years before birth

One-way between subjects ANOVAs were performed on baseline life satisfaction, separately for mothers and fathers. There were no significant differences in baseline satisfaction based on birth group for mothers [F(2, 5457) = 1.175, *p* = 0.31] nor in fathers [F(2, 4150) = 1.232, *p* = 0.29], for mean scores in each cohort see Tables 1 and 2. Results from the power analysis suggested that the ability to determine a small difference between term-born and MLP/LBW mothers or fathers was always sufficiently powered (> 0.90). The ability to determine a moderate difference for VP/VLBW mothers was also sufficiently powered at 0.84 but borderline in fathers at 0.77.

### Longitudinal changes in life satisfaction surrounding childbirth

**Figure 1:**
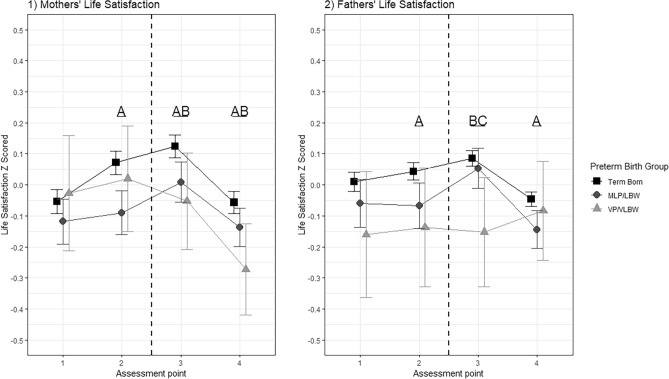
Estimated Marginal Means of Life Satisfaction for Mothers (left) and Fathers (right). Note. Letters indicate where the contrast analysis of marginal means was significant at *p* < 0.05, adjusted for multiple comparisons using the FDR correction. A = Term born vs MLP/LBW, B = Term born vs VP/VLBW, C = MLP/LBW vs VP/VLBW. The dashed line indicates the birth occurring. Assessment points 1, 2, 3 and 4 are 2 years before birth, 6 months before birth, 6 months after birth and 2 years after birth respectively.

For mothers and fathers separately, mixed models analysed changes in life satisfaction relative to birth. From these models, the estimated marginal means were calculated and contrasted (Tables 3, 4), and are displayed in Fig. 1.

**Table 3:** Within birthgroup changes in life satisfaction over time- marginal means contrast analysis.

Birth group	Assessment point contrast	Mothers' difference in satisfaction	Mothers adj. *p*. value	Fathers' difference in satisfaction	Fathers adj. *p*. value
Term Born	Two years before–Six months before	**−0.13**	**0.01**	**−**0.03	0.06
Term Born	Two years before–Six months after	**−0.18**	**0.01**	**−0.08**	**0.01**
Term Born	Two years before–two years after	0.00	0.89	**0.06**	**0.01**
Term Born	Six months before–Six months after	**−0.05**	**0.01**	**−0.04**	**0.01**
Term Born	Six months before–Two years after	**0.13**	**0.01**	**0.09**	**0.01**
Term Born	Six Months after–Two years after	**0.18**	**0.01**	**0.13**	**0.01**
MLP/LBW	Two years before–Six months before	**−**0.03	0.57	0.01	0.87
MLP/LBW	Two Years Before – Six months After	**−0.13**	**0.01**	**−0.11**	**0.02**
MLP/LBW	Two years before–Two years after	0.02	0.64	0.08	0.08
MLP/LBW	Six months before–Six months after	**−0.10**	**0.01**	**−0.12**	**0.01**
MLP/LBW	Six Months before–Two years after	0.05	0.30	0.08	0.08
MLP/LBW	Six Months after–Two years after	**0.15**	**0.01**	**0.20**	**0.01**
VP/VLBW	Two years before–Six months before	**−**0.05	0.79	**−**0.02	0.94
VP/VLBW	Two years before–Six months after	0.03	0.80	**−**0.01	0.94
VP/VLBW	Two years before–Two years after	**0.25**	**0.03**	**−**0.08	0.94
VP/VLBW	Six months before–Six months after	0.07	0.67	0.01	0.94
VP/VLBW	Six months before–Two years after	**0.29**	**0.01**	**−**0.05	0.94
VP/VLBW	Six months after–Two years after	**0.22**	**0.03**	**−**0.07	0.94

*MLP/LBW moderately or late preterm or low birthweight, *VP/VLBW* very preterm or very low birthweight. Contrasts are adjusted for multiple comparison using the false discovery rate adjustment. Bold indicates significance at p <0.05.

**Table 4:** Differences in birth group life satisfaction over time- marginal means contrast analysis.

Birth group contrast	Assessment point	Mothers’ difference in life satisfaction	Mothers adj. *p* value	Fathers’ difference in life satisfaction	Fathers adj.*p* value	
Term Born—MLP/LBW	1 (Two years before)	0.06	0.30	0.07	0.16	
Term Born—VP/VLBW	1 (Two years before)	-0.03	0.75	0.17	0.16	
MLP/LBW—VP/VLBW	1 (Two years before)	-0.09	0.55	0.10	0.36	
Term Born—MLP/LBW	2 (Six months before)	0.16	**< 0.001**	0.11	**0.02**	
Term Born—VP/VLBW	2 (Six months before)	0.05	0.57	0.18	0.10	
MLP/LBW—VP/VLBW	2 (Six months before)	-0.11	0.35	0.07	0.50	
Term Born—MLP/LBW	3 (Six months after)	0.11	**0.001**	0.03	0.39	
Term Born—VP/VLBW	3 (Six months after)﻿	0.17	**0.04**	0.24	**0.03**	
MLP/LBW—VP/VLBW	3 (Six months after)﻿	0.06	0.46	0.21	**0.05**	
Term Born—MLP/LBW	4 (Two years after)	0.08	**0.02**	0.10	**0.01**	
Term Born—VP/VLBW	4 ﻿(Two years after)	0.21	**0.01**	0.04	0.65	
MLP/LBW—VP/VLBW	4 ﻿(Two years after)	0.13	0.09	-0.06	0.65	

*MLP/LBW moderately or late preterm or low birthweight, *VP/VLBW* very preterm or very low birthweight. Contrasts are adjusted for multiple comparison using the false discovery rate adjustment. Bold indicates significance at *p* < 0.05.

The mixed model indicated that relative to their baseline life satisfaction two years before birth, scores for mothers of term-borns rose at assessment two and to their highest at assessment three, those six months prior to and post birth, (increase in Z score = 0.13 and 0.18, both *p* =  < 0.001) but then largely returned to baseline levels for the last assessment at two years post birth (increase in Z score = 0.01, *p* = 0.89), see Fig. 1.1 and Table 3. For mothers of MLP/LBW, life satisfaction also peaked at six months post birth, being 0.13Z higher than baseline (*p* = 0.01*)* but then also returned back to baseline levels at two years post birth. Finally, mothers of VP/VLBWs showed no increase in life satisfaction from baseline to six months post birth but then demonstrated a significant decline in life satisfaction at two years post birth relative to the three other assessments (decreases in Z scores all > 0.22, all *p* < *0.05).* Regarding contrasts of estimated marginal means by birth groups at the same assessments, mothers of term-borns had significantly higher life satisfaction than mothers of MLP/LBWs at assessments two, three and four, Fig. 1.1/Table 4. Additionally, mothers of term-borns had significantly higher life satisfaction than mothers of VP/VLBWs at assessments three and four, with the largest z difference equalling 0.21, Fig. 1.1/Table 4.

In the analysis of fathers, relative to baseline life satisfaction, scores for fathers of term-borns significantly rose in the third assessment but then significantly fell in the fourth assessment (differences in Z scores = 0.08 and −0.06, both *p* ≤ 0.001), see Fig. 1.2 and Table 3. For father of MLP/LBWs, life satisfaction also peaked at six months post birth, being 0.11Z higher than baseline (*p* = 0.02*)* but then also returned back to baseline levels at two years post birth. Finally, fathers of VP/VLBWs showed no significant increases or decreases in life satisfaction across the four assessment points. Regarding contrasts of birth groups, fathers of term-borns had significantly higher life satisfaction than fathers of MLP/LBWs at assessments two and four. Fathers of VP/VLBWs temporarily had lower life satisfaction than the two other birth groups at assessment three, with the largest difference equalling 0.24, see Fig. 1.2/ Table 4.

**Figure 2:**
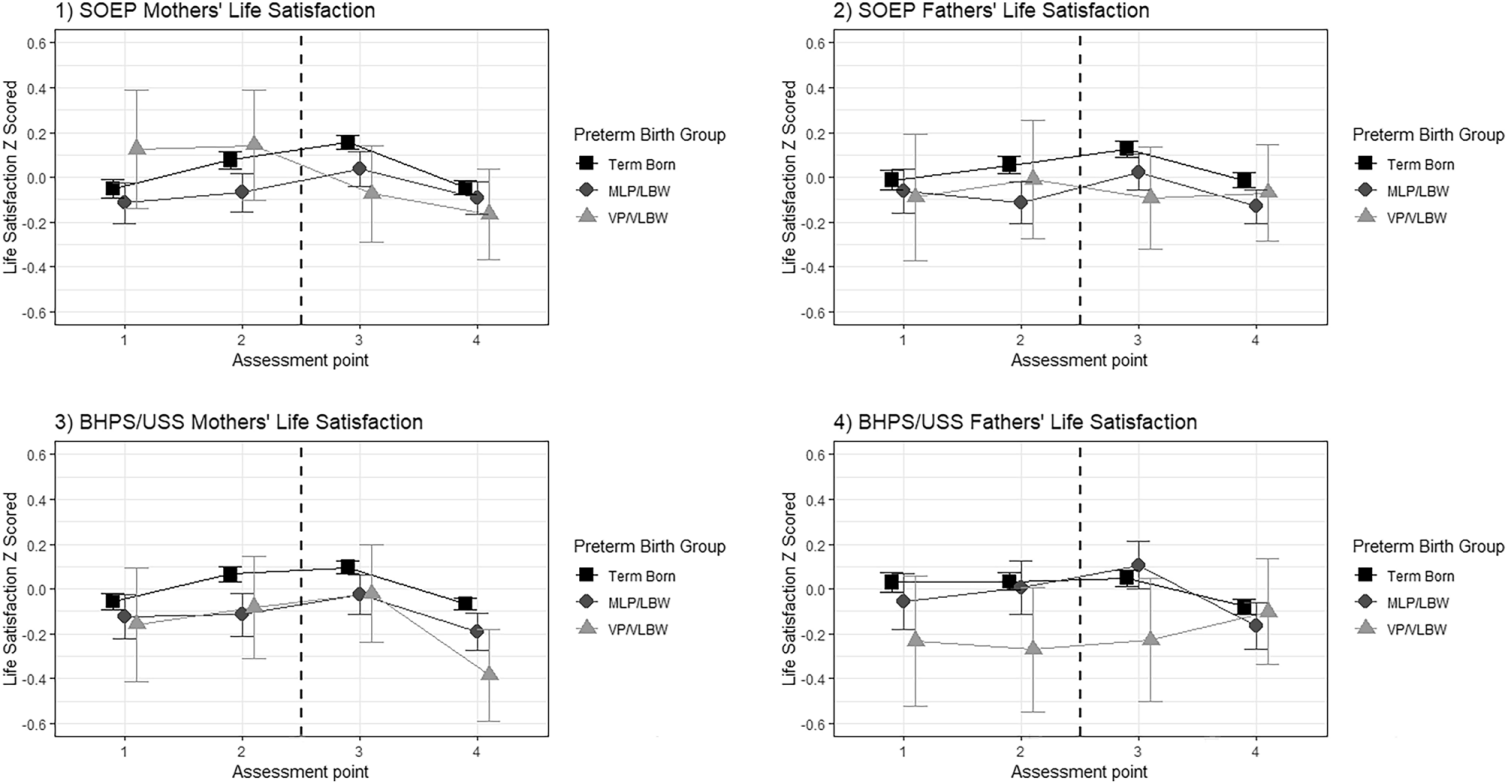
Estimated Marginal Means of Life Satisfaction for Each Cohort Separately and for Mothers (left) and Fathers (right). Note. The dashed line indicates the birth occurring. Assessment points 1, 2, 3 and 4 are 2 years before birth, 6 months before birth, 6 months after birth and 2 years after birth respectively.

Analyses were also performed separately by study, after the removal of specific subsamples, and after the inclusion of covariates, see Supplementary Tables S3−S6 and Fig. 2. Results appeared largely robust and consistent across studies; while lower parental education levels and the child having a long-term illness were particularly consistent predictors of lower life satisfaction, their addition resulted in minimal confounding of the association of birth group or assessment point with life satisfaction.

## Discussion

This study investigated changes in parents’ life satisfaction prior to and following the birth of term-born, MLP/LBW, or VP/VLBW infants. Consistently, no differences in life satisfaction between birth groups were found at baseline, for both mothers and fathers. Regarding subsequent differences between child birth groups, in mothers’ data, those with VP/VLBW infants showed a significant decline in life satisfaction in both assessments after birth relative to mothers of term borns. Furthermore, mothers of MLP/LBW infants had a smaller rise in life satisfaction in the assessment prior to birth and then remained consistently lower than mothers of term-born infants. Generally, while mothers appeared to be more affected by the birth than fathers, marginal mean differences in all analyses were relatively small, with Z score differences less than 0.25.

The first aim of the current study was to investigate life satisfaction before pregnancy. A major limitation of past cohort studies is that most started their recruitment at birth or later, limiting the ability to determine whether poorer wellbeing for parents of preterms only occurs after the birth happens. As research has linked increased prenatal stress with preterm birth^24^, it was plausible that the life satisfaction of the future parents of MLP/LBW or VP/VLBW infants would already be lower than those of future parents of term-borns. This finding would have been in concordance with the finding that postpartum depression is strongly associated with depression prior to pregnancy^30^. However, no significant differences in life satisfaction before the pregnancy based on future parental birth group were found, indicating that subsequent differences after birth cannot be simply attributed to a long standing, pre-existing difference.

The second aim was to investigate parents’ life satisfaction following birth, with our results showing smaller effect sizes when compared to past studies of preterm or LBW children that have used different recruitment strategies^6^. SOEP and BHPS/USS are household panel samples that recruit households and subsequently follow all household members annually. This contrasts to birth cohorts of preterm individuals that specifically recruit infants requiring admission to NICU^38^. Thus, the MLP/LBW or VP/VLBW infants in this analysis may reflect the parental life satisfaction for parents of all preterm/LBW infants, rather than a selective subsample with greater pathology. For example, the average gestation of the VP/VLBW sample was approximately 31 weeks, whereas past studies using cohort data often have lower average gestations, at approximately 27 or 28 weeks^6,10^. This may partly explain why our largest standardised difference of 0.24, measured six months post birth when differences between groups may be expected to be particularly pronounced, is somewhat smaller than the 0.34 effect size they reported at ages two, seven, and 13 years post birth^6^. While our effects were smaller, a noticeable difference in life satisfaction was found for mothers of VP/VLBW and term born infants at both assessments after birth, with VP/VLBW fathers also showing a temporary significant difference relative to other fathers in the assessment after birth. This is particularly important when interventions to support parents of preterms are considered, especially when considered in tandem with the fact that no pre-existing difference two years before birth were found. As the change in life satisfaction is concordant with the birth occurring, and found for both mothers and fathers of VP/VLBW infants, it suggests that support can be specifically focused after the birth occurs. This quantitative analysis therefore supports previous reports based on qualitative interviews of mothers of extremely preterm infants^39^.

Moreover, we investigated differences between mothers and fathers and consistency of findings across studies. The finding that life satisfaction also rises for UK mothers of term-borns in the years directly before and after birth, supports past findings only using the SOEP dataset^20^. It was also found that fathers showed only a small increase in the year after the birth while mothers reported a larger increase in life satisfaction in both assessments nearest to birth, corroborating past findings of larger effects for mothers^4^. Finally, the inclusion of multiple covariates did not consistently change our main findings regarding preterm birth and life satisfaction. These analyses show that, apart from preterm birth, lower education levels and having a child with a long-term illness are also associated with lower life satisfaction. Thus, when considering the targeting of intervention or identifying those most at risk, these parents appear to be more likely to require support.

When considering the strengths of the current study, the longitudinal approach utilising prospective measurements starting before pregnancy until two years after birth is a particular and unique strength. We could determine if differences in life satisfaction according to prematurity or LBW emerged after birth or whether they were pre-existing. Second, we were able to use two large samples, from two different countries but still using very similar methodology. This allowed for the robustness of our results to be considered. Third, the ability to consider a number of covariates demonstrated that the findings were not confounded by socio-environmental factors that have been previously associated with both preterm birth^23^ and life satisfaction^17^.

Limitations of the current study were that rather than relying on hospital records, information on birthweight and gestational age were parent reported, which may be subject to error. However, evidence suggests that a year after birth, over 90% of parents can accurately recall their child’s birthweight within 100 g^40^ or a week of gestation^41^. While gestation or birthweight are likely to be accurately reported, it may be a proxy measure of a factor that has a greater influence on parent’s life satisfaction, the child’s health. Past research has found that the association between preterm birth and parental wellbeing is mediated by neonatal morbidity and later child functioning^42^. Thus, future research may investigate the life satisfaction prior to and following childbirth that results in either admission to NICU specifically or results in long term morbidity for the child. In our study, information on morbidity was limited to a binary variable of whether the child had a health problem; more detailed variables could not be harmonized across the samples and for single-cohort-analyses the statistical power was lacking due to insufficient sample size. Another potential limitation is the use of a single-item life satisfaction measure rather than other measures of mental health or wellbeing, as it may be affected by social desirability bias^43^. However, past research indicates life satisfaction and mental health demonstrate a strong reciprocal relationship^18,19,44^, with mental health problems a larger contributor than income for explaining variance in life satisfaction^17^.

To conclude, the findings from two panel studies indicate that while parents of term-born infants show a rise in life satisfaction in the year directly before or after birth, this effect is less pronounced for parents of MLP/LBW and VP/VLBW infants. While mothers showed larger changes in life satisfaction surrounding birth, fathers of different birth groups showed less variation over time, with the differences across birth groups largely stable over time. Overall, while effect sizes were small, the relatively lower life satisfaction of mothers of VP/VLBWs infants at six months and two years after birth should be particularly noted and psychosocial support provided.

### Supplementary Information

Supplementary Information.

## Data Availability

The datasets generated and/or analysed during the current study are available in the UK data service and DIW repositories, (https://www.understandingsociety.ac.uk/ and https://www.diw.de/en/diw_01.c.615551.en/research_infrastructure__socio-economic_panel__soep.html).

## Abbreviations

BHPS: British Household Panel Study
USS: Understanding Society Study
SOEP: The German Socio-Economic Panel
VP/VLBW: Very Preterm or Very low Birthweight
MLP/LBW: Moderately/Late Preterm or Low Birthweight
