# Supplementary material for: Parents’ life satisfaction prior to and following preterm birth
Source: Sci Rep. 2023 Dec 1;13:21233. doi: 10.1038/s41598-023-48582-8 (PMC10692203; doi:10.1038/s41598-023-48582-8)
Supplement: Supplementary file 1 — Supplementary Information. [file 41598_2023_48582_MOESM1_ESM.docx]

Parents’ life satisfaction prior to and following preterm birth: Supplementary Material

Robert EVES PhD* ^1,2,^, Nicole BAUMANN PhD ^3,4^, Ayten BILGIN PhD ^5^, Daniel SCHNITZLEIN PhD^6,7^, David RICHTER PhD ^8^, Dieter WOLKE PhD Dr h.c. mult^2^, and Sakari LEMOLA PhD ^1^

**Supplementary Table S1 German’s SOEP data- extended demographics**

|  | **Fathers** | | | **Mothers** | | |
| --- | --- | --- | --- | --- | --- | --- |
|  | **Term Born (N=4079)** | **Moderately/ Late preterm or Low Birthweight (N=750)** | **Very Preterm/ Very Low Birthweight (N=94)** | **Term Born (N=4754)** | **Moderately/ Late preterm or Low Birthweight (N=865)** | **Very**  **Preterm/**  **Very Low**  **Birthweight (N=107)** |
| **Weight of Child at Birth (g)** |  |  |  |  |  |  |
| Mean (SD) | 3460 (451) | 2740 (621) | 1550 (779) | 3450 (452) | 2720 (609) | 1600 (820) |
| Missing | 16 (0%) | 12 (2%) | 0 (0%) | 22 (1%) | 11 (1%) | 0 (0%) |
| **Gestational Age (weeks)** |  |  |  |  |  |  |
| Mean (SD) | 39.7 (1.39) | 35.7 (1.78) | 30.0 (3.48) | 39.7 (1.40) | 35.7 (1.81) | 30.2 (3.64) |
| **Child’s Sex** |  |  |  |  |  |  |
| Male | 2082 (51%) | 386 (52%) | 50 (53%) | 2426 (51%) | 439 (51%) | 57 (53%) |
| Female | 1997 (49%) | 364 (49%) | 44 (47%) | 2328 (49%) | 426 (49%) | 50 (47%) |
| **Multiple Birth** |  |  |  |  |  |  |
| Yes (twin, triplet, etc.) | 20 (1%) | 79 (11%) | 19 (20%) | 26 (1%) | 82 (10%) | 22 (20%) |
| No (singleton) | 4059 (100%) | 671 (90%) | 75 (80%) | 4728 (100%) | 783 (91%) | 85 (80%) |
| **First Child^1^** |  |  |  |  |  |  |
| No | 1 (0%) | 117 (16%) | 16 (17%) | 1 (0%) | 134 (16%) | 17 (16%) |
| Yes | 4078 (100%) | 633 (84%) | 78 (83%) | 4753 (100%) | 731 (85%) | 90 (84%) |
|  |  |  |  |  |  |  |
| **Child’s Health** |  |  |  |  |  |  |
| Confirmed health condition | 116 (3%) | 41 (6%) | 15 (16%) | 146 (3%) | 51 (6%) | 15 (14%) |
| No confirmed health condition | 2852 (70%) | 426 (57%) | 47 (50%) | 3416 (72%) | 528 (61%) | 59 (55%) |
| Missing | 1111 (27%) | 283 (38%) | 32 (34%) | 1192 (25%) | 286 (33%) | 33 (31%) |
| **Age of Parent** |  |  |  |  |  |  |
| Mean (SD) | 34.0 (6.54) | 34.5 (6.95) | 33.8 (7.17) | 30.6 (5.84) | 30.5 (6.03) | 31.0 (5.52) |
| **Marital Status** |  |  |  |  |  |  |
| Legally Married/Civil Partnership | 2851 (69.9%) | 579 (77.2%) | 65 (69.1%) | 3024 (63.6%) | 577 (66.7%) | 67 (62.6%) |
| Legally Not Married/Civil Partnership | 1207 (29.6%) | 168 (22.4%) | 29 (30.9%) | 1705 (35.9%) | 281 (32.5%) | 40 (37.4%) |
| Missing | 21 (0.5%) | 3 (0.4%) | 0 (0%) | 25 (0.5%) | 7 (0.8%) | 0 (0%) |
| **Household Net Income per Month (Euros)** |  |  |  |  |  |  |
| Mean (SD) | 2980 (16100) | 2460 (1600) | 2430 (1470) | 2860 (14900) | 2380 (1520) | 2260 (1400) |
| Missing | 211 (5%) | 33 (4%) | 5 (5%) | 228 (5%) | 45 (5%) | 8 (8%) |
| **Parental Education Level** |  |  |  |  |  |  |
| 1) ISCED Low | 614 (15%) | 217 (29%) | 23 (25%) | 810 (17%) | 269 (31%) | 26 (24%) |
| 2) ISCED Medium | 2004 (49%) | 306 (41%) | 44 (47%) | 2443 (51%) | 356 (41%) | 55 (51%) |
| 3) ISCED High | 1328 (33%) | 192 (26%) | 23 (25%) | 1358 (29%) | 208 (24%) | 23 (22%) |
| Missing | 133 (3%) | 35 (5%) | 4 (4%) | 143 (3%) | 32 (4%) | 3 (3%) |
| **Migrant Background** |  |  |  |  |  |  |
| Migrant Background | 1554 (38%) | 394 (53%) | 37 (39%) | 1805 (38%) | 413 (48%) | 46 (43%) |
| No Migrant Background | 2525 (62%) | 356 (48%) | 57 (61%) | 2949 (62%) | 452 (52%) | 61 (57%) |

^1^The first child born while the parent is participating in the study

**Supplementary Table S2: UK’s BHPS and USS Data- extended demographics**

|  | **Fathers** | | | **Mother** | | |
| --- | --- | --- | --- | --- | --- | --- |
|  | **Term Born (N=4096)** | **MLP/LBW (N=441)** | **VP/VLBW (N=78)** | **Term Born (N=5665)** | **MLP/ LBW (N=627)** | **VP/VLBW (N=105)** |
| **Weight Of Child At Birth(g)** |  |  |  |  |  |  |
| Mean (SD) | 3490 (507) | 2380 (452) | 1230 (400) | 3470 (506) | 2380 (440) | 1260 (435) |
| Missing | 52 (1.3%) | 1 (0.2%) | 0 (0%) | 67 (1.2%) | 2 (0.3%) | 0 (0%) |
| **Gestational Age (weeks)** |  |  |  |  |  |  |
| Mean (SD) | 40.1 (1.31) | 36.4 (2.27) | 31.4 (4.68) | 40.1 (1.33) | 36.4 (2.28) | 31.7 (4.69) |
| Missing | 2 (0.0%) | 0 (0%) | 0 (0%) | 5 (0.1%) | 1 (0.2%) | 0 (0%) |
| **Child’s Sex** |  |  |  |  |  |  |
| Male | 2120 (51.8%) | 225 (51.0%) | 37 (47.4%) | 2909 (51.4%) | 316 (50.4%) | 53 (50.5%) |
| Female | 1976 (48.2%) | 216 (49.0%) | 41 (52.6%) | 2756 (48.6%) | 311 (49.6%) | 52 (49.5%) |
| **Multiple Birth** |  |  |  |  |  |  |
| Yes (twin, triplet, etc.) | 16 (0.4%) | 46 (10.4%) | 11 (14.1%) | 21 (0.4%) | 63 (10.0%) | 13 (12.4%) |
| No (singleton) | 4080 (99.6%) | 395 (89.6%) | 67 (85.9%) | 5644 (99.6%) | 564 (90.0%) | 92 (87.6%) |
| **First Child^1^** |  |  |  |  |  |  |
| Yes | 4080 (99.6%) | 358 (81.2%) | 64 (82.1%) | 5646 (99.7%) | 498 (79.4%) | 84 (80.0%) |
| No | 16 (0.4%) | 83 (18.8%) | 14 (17.9%) | 19 (0.3%) | 129 (20.6%) | 21 (20.0%) |
| **Child’s Health** |  |  |  |  |  |  |
| Confirmed health condition | 516 (12.6%) | 59 (13.4%) | 22 (28.2%) | 666 (11.8%) | 89 (14.2%) | 24 (22.9%) |
| No confirmed health condition | 1373 (33.5%) | 174 (39.5%) | 23 (29.5%) | 1839 (32.5%) | 247 (39.4%) | 36 (34.3%) |
| Missing | 2207 (53.9%) | 208 (47.2%) | 33 (42.3%) | 3160 (55.8%) | 291 (46.4%) | 45 (42.9%) |
| **Age of Parent** |  |  |  |  |  |  |
| Mean (SD) | 32.0 (6.73) | 32.5 (6.66) | 32.4 (6.10) | 28.6 (6.13) | 28.7 (6.16) | 28.7 (5.82) |
| **Marital status** |  |  |  |  |  |  |
| Legally Married/Civil Partnership | 2371 (57.9%) | 279 (63.3%) | 50 (64.1%) | 2682 (47.3%) | 305 (48.6%) | 55 (52.4%) |
| Legally Not Married/Civil Partnership | 1692 (41.3%) | 158 (35.8%) | 28 (35.9%) | 2889 (51.0%) | 303 (48.3%) | 47 (44.8%) |
| Missing | 33 (0.8%) | 4 (0.9%) | 0 (0%) | 94 (1.7%) | 19 (3.0%) | 3 (2.9%) |
| **total household net income - no deductions** |  |  |  |  |  |  |
| Mean (SD) | 2820 (2150) | 2790 (1910) | 2910 (2780) | 2650 (2060) | 2560 (1580) | 2470 (1830) |
| Median [Min, Max] | 2440 [-2550, 46600] | 2390 [0, 24500] | 2260 [144, 18300] | 2290 [-5410, 46600] | 2230 [0, 12100] | 2130 [144, 12900] |
| Missing | 4 (0.1%) | 0 (0%) | 0 (0%) | 7 (0.1%) | 2 (0.3%) | 1 (1.0%) |
| **ISCED_3_Levels** |  |  |  |  |  |  |
| ISCED High | 1323 (32.3%) | 152 (34.5%) | 29 (37.2%) | 1902 (33.6%) | 184 (29.3%) | 28 (26.7%) |
| ISCED Low | 620 (15.1%) | 73 (16.6%) | 17 (21.8%) | 729 (12.9%) | 102 (16.3%) | 18 (17.1%) |
| ISCED Medium | 2002 (48.9%) | 199 (45.1%) | 29 (37.2%) | 2815 (49.7%) | 320 (51.0%) | 54 (51.4%) |
| Missing | 151 (3.7%) | 17 (3.9%) | 3 (3.8%) | 219 (3.9%) | 21 (3.3%) | 5 (4.8%) |
| **Migrant Background** |  |  |  |  |  |  |
| Migrant Background | 1232 (30.1%) | 158 (35.8%) | 34 (43.6%) | 1822 (32.2%) | 247 (39.4%) | 51 (48.6%) |
| Missing/inapplicable/refusal | 291 (7.1%) | 40 (9.1%) | 5 (6.4%) | 438 (7.7%) | 33 (5.3%) | 7 (6.7%) |
| No Migrant Background | 2573 (62.8%) | 243 (55.1%) | 39 (50.0%) | 3405 (60.1%) | 347 (55.3%) | 47 (44.8%) |

^1^The first child born while the parent is participating in the study

### **Supplementary Table S3 SOEP mothers: standard model (left), without refugee sub-samples (middle) and with covariates (right)**

|  | **Life Satisfaction Z Score** | | | **Life Satisfaction Z Score** | | | **Life Satisfaction Z Score** | | |
| --- | --- | --- | --- | --- | --- | --- | --- | --- | --- |
| *Predictors* | *Estimates* | *CI* | *p* | *Estimates* | *CI* | *p* | *Estimates* | *CI* | *p* |
| (Intercept) | -0.05 | -0.09 – -0.01 | **0.012** | -0.07 | -0.12 – -0.02 | **0.003** | -0.11 | -0.37 – 0.14 | 0.385 |
| Assessment Point: 2 | 0.13 | 0.08 – 0.17 | **<0.001** | 0.13 | 0.08 – 0.18 | **<0.001** | 0.15 | 0.09 – 0.21 | **<0.001** |
| Assessment Point: 3 | 0.21 | 0.16 – 0.25 | **<0.001** | 0.21 | 0.16 – 0.25 | **<0.001** | 0.21 | 0.16 – 0.27 | **<0.001** |
| Assessment Point: 4 | 0.01 | -0.04 – 0.05 | 0.801 | 0.01 | -0.04 – 0.06 | 0.690 | -0.03 | -0.09 – 0.03 | 0.391 |
| MLP/LBW | -0.06 | -0.16 – 0.04 | 0.222 | -0.07 | -0.18 – 0.05 | 0.269 | -0.00 | -0.14 – 0.13 | 0.946 |
| VP/VLBW | 0.18 | -0.09 – 0.45 | 0.196 | 0.29 | -0.01 – 0.60 | 0.062 | 0.26 | -0.09 – 0.61 | 0.149 |
| Assessment Point: 2*MLP/LBW | -0.08 | -0.19 – 0.03 | 0.149 | -0.06 | -0.19 – 0.07 | 0.397 | -0.16 | -0.31 – -0.01 | **0.032** |
| Assessment Point: 3*MLP/LBW | -0.06 | -0.16 – 0.05 | 0.291 | -0.05 | -0.17 – 0.07 | 0.450 | -0.11 | -0.24 – 0.03 | 0.130 |
| Assessment Point: 4*MLP/LBW | 0.02 | -0.09 – 0.12 | 0.758 | 0.03 | -0.09 – 0.15 | 0.610 | -0.02 | -0.16 – 0.12 | 0.777 |
| Assessment Point: 2*VP/VLBW | -0.11 | -0.41 – 0.19 | 0.471 | -0.17 | -0.51 – 0.17 | 0.318 | -0.08 | -0.47 – 0.31 | 0.690 |
| Assessment Point: 3*VP/VLBW | -0.41 | -0.69 – -0.12 | **0.005** | -0.39 | -0.72 – -0.07 | **0.017** | -0.40 | -0.77 – -0.03 | **0.034** |
| Assessment Point: 4*VP/VLBW | -0.30 | -0.58 – -0.01 | **0.043** | -0.33 | -0.64 – -0.01 | **0.042** | -0.20 | -0.57 – 0.17 | 0.291 |
| Household Income |  |  |  |  |  |  | 0.01 | -0.01 – 0.02 | 0.230 |
| ISCED Medium (ref: ISCED low) |  |  |  |  |  |  | 0.15 | 0.07 – 0.22 | **<0.001** |
| ISCED High (ref: ISCED low) |  |  |  |  |  |  | 0.35 | 0.26 – 0.43 | **<0.001** |
| Age of Parent |  |  |  |  |  |  | -0.00 | -0.01 – 0.00 | 0.144 |
| First Child (ref: non first child) |  |  |  |  |  |  | 0.01 | -0.14 – 0.16 | 0.899 |
| Multiple Birth (ref: singleton) |  |  |  |  |  |  | 0.08 | -0.10 – 0.25 | 0.384 |
| No Migrant Background (ref: migrant background) |  |  |  |  |  |  | -0.05 | -0.11 – -0.00 | **0.047** |
| Sibling Born within Assessment 1 Timeframe |  |  |  |  |  |  | 0.04 | -0.23 – 0.31 | 0.762 |
| Sibling Born within Assessment 2 Timeframe |  |  |  |  |  |  | 0.43 | -0.70 – 1.55 | 0.457 |
| Sibling Born within Assessment 3 Timeframe |  |  |  |  |  |  | -0.45 | -1.03 – 0.12 | 0.122 |
| Sibling Born within Assessment 4 Timeframe |  |  |  |  |  |  | 0.15 | 0.07 – 0.22 | **<0.001** |
| Marital Status (Not married, ref: Married |  |  |  |  |  |  | -0.20 | -0.25 – -0.16 | **<0.001** |
| Child Sex (ref: Male) |  |  |  |  |  |  | -0.01 | -0.06 – 0.04 | 0.788 |
| No child illness (ref: child has illness) |  |  |  |  |  |  | 0.15 | 0.03 – 0.27 | **0.014** |
| **Random Effects** | | | | | | | | | |
| σ2 | 0.54 | | | 0.50 | | | 0.49 | | |
| τ00 pid | 0.45 | | | 0.45 | | | 0.39 | | |
| ICC | 0.45 | | | 0.48 | | | 0.44 | | |
| N pid | 5726 | | | 4340 | | | 3995 | | |
| Observations | 14077 | | | 10425 | | | 8951 | | |
| Marginal R2 / Conditional R2 | 0.009 / 0.459 | | | 0.008 / 0.480 | | | 0.043 / 0.466 | | |

### **Supplementary Table S4 SOEP fathers, standard model (left), without refugee sub-samples (middle) and with covariates (right)**

|  | **Life Satisfaction Z Score** | | | **Life Satisfaction Z Score** | | | **Life Satisfaction Z Score** | | |
| --- | --- | --- | --- | --- | --- | --- | --- | --- | --- |
| *Predictors* | *Estimates* | *CI* | *p* | *Estimates* | *CI* | *p* | *Estimates* | *CI* | *p* |
| (Intercept) | -0.01 | -0.06 – 0.03 | 0.579 | -0.01 | -0.06 – 0.04 | 0.637 | 0.15 | -0.12 – 0.43 | 0.279 |
| Assessment Point: 2 | 0.07 | 0.02 – 0.12 | **0.008** | 0.07 | 0.02 – 0.13 | **0.008** | 0.08 | 0.02 – 0.14 | **0.011** |
| Assessment Point: 3 | 0.14 | 0.09 – 0.18 | **<0.001** | 0.12 | 0.07 – 0.17 | **<0.001** | 0.15 | 0.09 – 0.21 | **<0.001** |
| Assessment Point: 4 | -0.00 | -0.05 – 0.05 | 0.994 | 0.00 | -0.05 – 0.05 | 0.953 | -0.02 | -0.08 – 0.05 | 0.610 |
| MLP/LBW | -0.05 | -0.16 – 0.06 | 0.378 | -0.04 | -0.17 – 0.09 | 0.552 | -0.08 | -0.23 – 0.07 | 0.285 |
| VP/VLBW | -0.08 | -0.36 – 0.21 | 0.601 | -0.03 | -0.35 – 0.29 | 0.844 | 0.05 | -0.36 – 0.47 | 0.802 |
| Assessment Point: 2*MLP/LBW | -0.12 | -0.24 – 0.00 | 0.054 | 0.02 | -0.12 – 0.16 | 0.772 | -0.08 | -0.24 – 0.08 | 0.339 |
| Assessment Point: 3*MLP/LBW | -0.05 | -0.17 – 0.06 | 0.374 | -0.01 | -0.15 – 0.12 | 0.872 | -0.07 | -0.22 – 0.08 | 0.380 |
| Assessment Point: 4*MLP/LBW | -0.07 | -0.19 – 0.05 | 0.238 | -0.04 | -0.17 – 0.09 | 0.546 | -0.07 | -0.22 – 0.08 | 0.367 |
| Assessment Point: 2*VP/VLBW | 0.01 | -0.31 – 0.33 | 0.944 | -0.07 | -0.42 – 0.29 | 0.709 | -0.23 | -0.69 – 0.24 | 0.339 |
| Assessment Point: 3*VP/VLBW | -0.14 | -0.44 – 0.16 | 0.366 | -0.17 | -0.50 – 0.17 | 0.331 | -0.22 | -0.65 – 0.21 | 0.319 |
| Assessment Point: 4*VP/VLBW | 0.02 | -0.29 – 0.32 | 0.903 | -0.07 | -0.39 – 0.26 | 0.681 | -0.06 | -0.49 – 0.37 | 0.786 |
| Household Income (Z scored) |  |  |  |  |  |  | 0.01 | -0.00 – 0.02 | 0.164 |
| ISCED Medium (ref: ISCED low) |  |  |  |  |  |  | 0.16 | 0.07 – 0.25 | **<0.001** |
| ISCED High (ref: ISCED low) |  |  |  |  |  |  | 0.38 | 0.29 – 0.48 | **<0.001** |
| Age of Parent |  |  |  |  |  |  | -0.01 | -0.01 – -0.00 | **0.026** |
| First Child (ref: non first child) |  |  |  |  |  |  | -0.05 | -0.22 – 0.11 | 0.541 |
| Multiple Birth (ref: singleton) |  |  |  |  |  |  | 0.16 | -0.02 – 0.35 | 0.080 |
| No Migrant Background (ref: migrant background) |  |  |  |  |  |  | -0.05 | -0.11 – 0.01 | 0.111 |
| Sibling Born within Assessment 1 Timeframe |  |  |  |  |  |  | 0.34 | -0.75 – 1.44 | 0.538 |
| Sibling Born within Assessment 2 Timeframe |  |  |  |  |  |  | -1.11 | -1.83 – -0.39 | **0.003** |
| Sibling Born within Assessment 3 Timeframe |  |  |  |  |  |  | 0.10 | 0.02 – 0.18 | **0.013** |
| Sibling Born within Assessment 4 Timeframe |  |  |  |  |  |  | -0.16 | -0.21 – -0.10 | **<0.001** |
| Marital Status (Not married, ref: Married |  |  |  |  |  |  | 0.05 | -0.25 – 0.36 | 0.739 |
| Child Sex (ref: Male) |  |  |  |  |  |  | -0.05 | -0.11 – 0.00 | 0.058 |
| No child illness(ref: child has illness) |  |  |  |  |  |  | 0.06 | -0.07 – 0.19 | 0.362 |
| **Random Effects** | | | | | | | | | |
| σ^2^ | 0.55 | | | 0.45 | | | 0.46 | | |
| τ_00_ | 0.45 _pid_ | | | 0.44_pid_ | | | 0.39 | | |
| ICC | 0.45 | | | 0.49 | | | 0.46 | | |
| N | 4923 _pid_ | | | 3626 _pid_ | | | 3310 | | |
| Observations | 12057 | | | 8563 | | | 7302 | | |
| Marginal R^2^ / Conditional R^2^ | 0.005/ 0.452 | | | 0.004 / 0.493 | | | 0.037 / 0.474 | | |

### **Supplementary Table S5 BHPS/USS Mothers, standard model (left) and after including covariates (right):**

|  | **Life Satisfaction Z Score** | | | **Life Satisfaction Z Score** | | |
| --- | --- | --- | --- | --- | --- | --- |
| *Predictors* | *Estimates* | *CI* | *p* | *Estimates* | *CI* | *p* |
| (Intercept) | -0.06 | -0.09 – -0.02 | **0.002** | -0.22 | -0.50 – 0.07 | 0.142 |
| Assessment Point: 2 | 0.12 | 0.08 – 0.16 | **<0.001** | 0.10 | 0.04 – 0.16 | **0.001** |
| Assessment Point: 3 | 0.15 | 0.11 – 0.19 | **<0.001** | 0.21 | 0.15 – 0.27 | **<0.001** |
| Assessment Point: 4 | -0.01 | -0.05 – 0.03 | 0.573 | 0.01 | -0.06 – 0.07 | 0.846 |
| MLP/LBW | -0.07 | -0.17 – 0.04 | 0.212 | -0.01 | -0.17 – 0.14 | 0.863 |
| VP/VLBW | -0.10 | -0.35 – 0.16 | 0.452 | -0.03 | -0.38 – 0.31 | 0.853 |
| Assessment Point: -2*MLP/LBW | -0.11 | -0.24 – 0.01 | 0.067 | -0.13 | -0.31 – 0.05 | 0.147 |
| Assessment Point: 3*MLP/LBW | -0.06 | -0.18 – 0.06 | 0.355 | -0.07 | -0.24 – 0.11 | 0.436 |
| Assessment Point: 4*MLP/LBW | -0.06 | -0.18 – 0.06 | 0.323 | -0.08 | -0.26 – 0.09 | 0.367 |
| Assessment Point: 2*VP/VLBW | -0.05 | -0.34 – 0.25 | 0.755 | 0.03 | -0.37 – 0.43 | 0.876 |
| Assessment Point: 3*VP/VLBW | -0.02 | -0.30 – 0.27 | 0.905 | 0.09 | -0.32 – 0.49 | 0.668 |
| Assessment Point: 4*VP/VLBW | -0.21 | -0.49 – 0.07 | 0.145 | -0.04 | -0.45 – 0.37 | 0.858 |
| total household net income |  |  |  | 0.03 | 0.01 – 0.05 | **0.003** |
| ISCED Medium (ref: ISCED low) |  |  |  | 0.15 | 0.06 – 0.25 | **0.002** |
| ISCED High (ref: ISCED low) |  |  |  | 0.40 | 0.30 – 0.50 | **<0.001** |
| Parental Age |  |  |  | -0.01 | -0.01 – -0.00 | **0.004** |
| First Child (ref: non first child) |  |  |  | 0.08 | -0.10 – 0.26 | 0.367 |
| Multiple Birth (ref: singleton) |  |  |  | 0.12 | -0.11 – 0.35 | 0.289 |
| Migrant Background Missing/inapplicable/refusal  (ref: Migrant Background) |  |  |  | 0.16 | 0.02 – 0.30 | **0.022** |
| No Migrant Background (ref: Migrant Background) |  |  |  | 0.14 | 0.08 – 0.21 | **<0.001** |
| Sibling Born within Assessment 1 Timeframe |  |  |  | 0.08 | -0.23 – 0.40 | 0.602 |
| Sibling Born within Assessment 2 Timeframe |  |  |  | -0.47 | -1.78 – 0.83 | 0.476 |
| Sibling Born within Assessment 3 Timeframe |  |  |  | -0.30 | -1.03 – 0.44 | 0.432 |
| Sibling Born within Assessment 4 Timeframe |  |  |  | 0.09 | -0.01 – 0.19 | 0.069 |
| Marital Status (Not married, ref: Married |  |  |  | -0.18 | -0.24 – -0.12 | **<0.001** |
| Child Sex (ref: Male) |  |  |  | -0.01 | -0.07 – 0.05 | 0.771 |
| No child illness (ref:child has illness) |  |  |  | 0.09 | 0.02 – 0.16 | **0.011** |
| **Random Effects** | | | | | | |
| σ^2^ | 0.61 | | | 0.69 | | |
| τ_00_ | 0.39 | | | 0.34 | | |
| ICC | 0.39 | | | 0.33 | | |
| N | 6399 | | | 2695 | | |
| Observations | 16845 | | | 7754 | | |
| Marginal R^2^ / Conditional R^2^ | 0.008 / 0.396 | | | 0.045 / 0.364 | | |

### **Supplementary Table S6 BHPS/USS Fathers standard model (left) and after including covariates (right):**

|  | **Life Satisfaction Z Score** | | | **Life Satisfaction Z Score** | | |
| --- | --- | --- | --- | --- | --- | --- |
| *Predictors* | *Estimates* | *CI* | *p* | *Estimates* | *CI* | *p* |
| (Intercept) | 0.03 | -0.01 – 0.07 | 0.179 | -0.18 | -0.54 – 0.17 | 0.306 |
| Assessment Point: 2 | 0.00 | -0.05 – 0.05 | 0.907 | -0.04 | -0.11 – 0.04 | 0.324 |
| Assessment Point: 3 | 0.02 | -0.03 – 0.07 | 0.453 | 0.00 | -0.07 – 0.08 | 0.894 |
| Assessment Point: 4 | -0.11 | -0.15 – -0.06 | **<0.001** | -0.14 | -0.22 – -0.06 | **<0.001** |
| MLP/LBW | -0.08 | -0.21 – 0.05 | 0.213 | -0.10 | -0.30 – 0.10 | 0.315 |
| VP/VLBW | -0.26 | -0.56 – 0.03 | 0.082 | -0.20 | -0.61 – 0.22 | 0.346 |
| Assessment Point: 2*MLP/LBW | 0.06 | -0.09 – 0.21 | 0.449 | 0.15 | -0.08 – 0.38 | 0.189 |
| Assessment Point: 3*MLP/LBW | 0.15 | 0.00 – 0.29 | **0.048** | 0.21 | -0.02 – 0.43 | 0.068 |
| Assessment Point: 4*MLP/LBW | -0.01 | -0.15 – 0.14 | 0.916 | 0.07 | -0.15 – 0.29 | 0.539 |
| Assessment Point: 2*VP/VLBW | -0.04 | -0.38 – 0.30 | 0.812 | 0.31 | -0.17 – 0.79 | 0.206 |
| Assessment Point: 3*VP/VLBW | -0.02 | -0.36 – 0.33 | 0.930 | 0.27 | -0.23 – 0.77 | 0.288 |
| Assessment Point: 4*VP/VLBW | 0.24 | -0.09 – 0.56 | 0.149 | 0.34 | -0.14 – 0.81 | 0.164 |
| total household net income - no deductions |  |  |  | 0.12 | 0.08 – 0.16 | **<0.001** |
| ISCED Medium (ref: ISCED low) |  |  |  | 0.06 | -0.04 – 0.17 | 0.229 |
| ISCED High (ref: ISCED low) |  |  |  | 0.15 | 0.04 – 0.26 | **0.008** |
| Parental Age |  |  |  | -0.01 | -0.02 – -0.00 | **0.001** |
| First Child (ref: non first child) |  |  |  | 0.15 | -0.08 – 0.39 | 0.198 |
| Multiple Birth (ref: singleton) |  |  |  | -0.03 | -0.29 – 0.24 | 0.851 |
| Migrant Background Missing/inapplicable/refusal  (ref: Migrant Background) |  |  |  | 0.24 | 0.06 – 0.43 | **0.010** |
| No Migrant Background (ref: Migrant Background) |  |  |  | 0.24 | 0.16 – 0.32 | **<0.001** |
| Sibling Born within Assessment 1 Timeframe |  |  |  | -0.05 | -0.44 – 0.34 | 0.813 |
| Sibling Born within Assessment 2 Timeframe |  |  |  | -0.37 | -1.71 – 0.97 | 0.588 |
| Sibling Born within Assessment 3 Timeframe |  |  |  | 0.04 | -0.88 – 0.96 | 0.935 |
| Sibling Born within Assessment 4 Timeframe |  |  |  | 0.13 | 0.01 – 0.24 | **0.028** |
| Marital Status (Not married, ref: Married |  |  |  | -0.16 | -0.23 – -0.08 | **<0.001** |
| Child Sex (ref: Male) |  |  |  | 0.06 | -0.02 – 0.13 | 0.127 |
| No child illness (ref: child has illness) |  |  |  | 0.11 | 0.03 – 0.19 | **0.010** |
| **Random Effects** | | | | | | |
| σ^2^ | 0.60 | | | 0.70 | | |
| τ_00_ | 0.41 | | | 0.40 | | |
| ICC | 0.40 | | | 0.37 | | |
| N | 4620 | | | 2061 | | |
| Observations | 11788 | | | 5573 | | |
| Marginal R^2^ / Conditional R^2^ | 0.005 / 0.406 | | | 0.032 / 0.388 | | |
